# High Fat Rodent Models of Type 2 Diabetes: From Rodent to Human

**DOI:** 10.3390/nu12123650

**Published:** 2020-11-27

**Authors:** Nicole L. Stott, Joseph S. Marino

**Affiliations:** Laboratory of Systems Physiology, Department of Kinesiology, The University of North Carolina at Charlotte, Charlotte, NC 28223, USA; Nstott1@uncc.edu

**Keywords:** high-fat diet, metabolism, type 2 diabetes, insulin resistance, obesity, rodent models of type 2 diabetes

## Abstract

Poor dietary habits contribute to increased incidences of obesity and related co-morbidities, such as type 2 diabetes (T2D). The biological, genetic, and pathological implications of T2D, are commonly investigated using animal models induced by a dietary intervention. In spite of significant research contributions, animal models have limitations regarding the translation to human pathology, which leads to questioning their clinical relevance. Important considerations include diet-specific effects on whole organism energy balance and glucose and insulin homeostasis, as well as tissue-specific changes in insulin and glucose tolerance. This review will examine the T2D-like phenotype in rodents resulting from common diet-induced models and their relevance to the human disease state. Emphasis will be placed on the disparity in percentages and type of dietary fat, the duration of intervention, and whole organism and tissue-specific changes in rodents. An evaluation of these models will help to identify a diet-induced rodent model with the greatest clinical relevance to the human T2D pathology. We propose that a 45% high-fat diet composed of approximately one-third saturated fats and two-thirds unsaturated fats may provide a diet composition that aligns closely to average Western diet macronutrient composition, and induces metabolic alterations mirrored by clinical populations.

## 1. Introduction 

High fat diet (HFD) animal models utilize a variety of fat sources to mimic the typical Western diet, which in the U.S. population consists of ~70% more saturated fat than the recommended dietary guidelines [1]. A twelve-year National Health and Nutrition Examination Study (NHANES) study demonstrated that individuals consuming high amounts of carbohydrates, cholesterol, saturated fatty acids, polyunsaturated fats, monounsaturated fats, and protein have a greater propensity to develop glucose intolerance when compared to individuals with dietary patterns high in vitamin, mineral, and fiber content [2]. Saturated fatty acids (SFA), which are stored more readily in rodents and humans compared to monounsaturated and polyunsaturated fats, increase the risk of obesity [3]. Therefore, it is important to work towards the uniform formulation of a rodent diet that best represents human consumption, while promoting a similar type 2 diabetes (T2D) phenotype. 

HFD consumed *ab libitum* results in rodents exceeding typical daily caloric intake [4], and animal-based fats promote diet-induced obesity and insulin resistance better than vegetable-based fats in rats [5]. Commonly used saturated fatty acid sources include hydrogenated coconut oil, corn oil, lard, palmitic acid, and stearic acid [6,7]. Unsaturated fats, such as oleic acid and linoleic acid, are also utilized in some nutritional animal models [8]. HFD, even when isocalorically matched with a standard purified control diet, induces obesity in rodents through alterations in metabolic homeostasis and reduced physical activity levels [9]. HFD-induced phenotypes of T2D in rodents and humans share weight gain, hyperglycemia, hyperinsulinemia, insulin resistance, inflammatory cytokine secretion, and ectopic lipid accumulation [10]. However, diet-induced animal models employ marked differences in micronutrient and macronutrient composition, including a variety of saturated fats, resulting in significant inter-study variability [11,12,13].

Despite these variables, the chronic consumption of a HFD by rodents alters a variety of genes and/or receptors involved in metabolism, inflammation, oxidative stress, substrate transport, protein synthesis and modification, and transcriptional regulation [6,7,14,15,16]. Notably, the modification of such genes is tissue-specific [6]. A better understanding of tissue specific effects in response to dietary composition will provide guidance into the most appropriate HFD-induced animal model(s) to study the pathogenesis of T2D and associated phenotypic changes. 

Thorough review articles addressing the value and limitations of T2D animal models have been published [10,17,18]. Variable diet composition, rodent strain, intervention time-points, and duration of protocols further complicate the translatability of animal-based findings to the human disease state. Here, we summarize how the combination of percentage and type of fat with varying durations of intervention, affect the major insulin responsive tissues. These are important considerations to get closer to an animal model with high clinical relevance to the human disease.

## 2. Tissue-Specific Effects of HFD Models

### 2.1. Liver

*Mice*. The composition of saturated fatty acids in a HFD differentially affects adipose deposition within the liver and subsequently cellular and molecular signaling. Examples of saturated fats that adversely affect the liver of C57BL/6 male mice include corn oil, lard, and hydrogenated coconut oil (Table 1). The composition of HFD is responsible for the different effects in hepatic lipid storage. For example, a 45% HFD containing palm oil (high in saturated fat) or olive oil (low in saturated fat) increased hepatic triacylglycerol (TAG) content following an 8-week intervention in C57BL/6 mice [19]. In male and female C57BL/6 mice, 18 to 20 weeks of a 60% lard-based diet increased lipid accumulation and steatosis in the liver [20,21,22]. Other studies showed hepatic inflammation and fibrosis following only 8 weeks of a similar diet [23].

On a whole organism level, male and female mice developed insulin resistance and glucose intolerance in response to 45% [19] and 60% HFD [20,21,22]. However, the source of the fat must be considered [19] (Table 1). 

Inflammatory responses and β-oxidation gene changes in mice have been reported, particularly as a result of a HFD containing more than 45% fat. Inflammatory responses are a known etiology of T2D development in rodents and humans [16,28]. However, establishing similarities between species is difficult when models vary in the percentage of fat, 59% to 82%, and duration, 12 to 36 weeks [6,29]. Eighteen to 20 weeks of a 60% lard-based diet increased cellular and biochemical markers of hepatic inflammation in male [20,21], but not female mice [22]. As the percentage of saturated fat increases, so does liver dysfunction. Interestingly an extremely HFD, 82% lard, caused hepatic lipid deposition accompanied by a transient increase in the expression of genes regulating fatty acid oxidation and synthesis after just 2 weeks [29]. By 4 weeks of consuming such a diet, gene expression patterns changed to reflect 2 to 4-fold increases adipogenesis (PPARγ and fatty acid binding protein) and lipid deposition (CD36) [29]. Not surprisingly, as the percentage of HFD increased, the inflammatory response followed the same trend. These studies suggest that excessive high fat content may cause a rapid transient pattern in hepatic fatty acid metabolism. Due to the rapid changes, it is plausible that many of the cellular and molecular events that alter hepatic lipid accumulation may be missed by studies focusing on prolonged consumption, especially when employing very high fat content. Due to the more progressive phenotype of lipid accumulation in human liver, such studies may contribute little to the understanding of the human phenotype. 

*Rats*. Four weeks of a 45% lard-based diet caused hepatic steatosis, increased liver mass and increased hepatic TAG content in Wister rats [25] (Table 1). Changes in liver characteristics were accompanied by obesity and reduced insulin sensitivity, despite no evidence of hyperglycemia [25]. This more mild phenotype may be the result of a short diet intervention period and/or modest concentration of fat. In response to diets containing higher concentrations of SFA, a more severe phenotype emerges. Sprague–Dawley rats developed hepatic insulin resistance, fatty liver, inflammation, and necrosis following consumption of a 58% butter [27] or 65% lard-based diet [30]. Under both dietary conditions, a severer whole body T2D phenotype emerged [27,30] (Table 1). 

The composition of fat within a diet may be just as important as the percentage and treatment duration. While a 45.5% HFD composed of SFA, monounsaturated fatty acids (MUFA), and polyunsaturated fatty acids (PUFA) did not cause an increase in body mass compared to control fed animals, liver mass and blood glucose were elevated after 15 weeks [31]. Such findings are similar to those reported in mice, such that higher concentrations of MUFA and PUFA mixtures produce a more mild phenotype [19] (Table 1).

### 2.2. Adipose

*Mice*. Insulin promotes lipogenesis in adipose tissue, reducing glycerol and fatty acid availability. Insulin resistant adipose tissue impairs glucose disposal and increases lipolysis, promoting fatty acid and glycerol availability for hepatic gluconeogenesis [32]. When unable to regulate hepatic glucose production properly, hyperglycemia coincides with lipid accumulation in adipose depots and peripheral tissues. 

Saturated and unsaturated fat differentially effect the metabolic and immune responses in adipose tissue. A 45% HFD enriched with palmitic acid (SFA) induced adipose tissue hypertrophy, but a 45% HFD, enriched with oleic acid (MUFA), caused adipose tissue hyperplasia [33] (Table 2). Insulin sensitivity was improved, but not normalized, in mice fed monounsaturated fat [34]. Improved insulin sensitivity was partially a result of adipose-mediated inflammatory signaling. Adipose tissue macrophages secrete pro-inflammatory mediators, such as tumor necrosis factor-alpha (TNF-α), interleukin-6 (IL-6), and monocyte chemoattractant protein-1 (MCP-1), which correlate with adipocyte cell death [35] (Table 2). A 45% HFD enriched with saturated fats exacerbated the inflammatory response within adipose tissue, evidenced by increased TNF-α and IL-6 gene expression and reduced insulin sensitivity [36]. Furthermore, 31 weeks of a 42% HFD composed of SFA and MUFA caused white adipose tissue inflammation, reduced insulin signaling, and reduced the capacity for mitochondrial biogenesis and function in male mice [34]. However, when the fat source was based on mono or polyunsaturated fats, the inflammatory response was mitigated, and insulin responsiveness restored [36]. 

An HFD of 60% caused a more severe inflammatory response [35]. Progressive increases in adipocyte size were followed by cell death with increased fat pad weight through 20 weeks [35]. This phase of adipose tissue remolding was largely driven by macrophage infiltration [35] and may be associated with adipose hyperplasia to account for adipocyte death. Following as little as 1 week of a 60% lard-based HFD, pro-inflammatory macrophages (M1-like macrophages) were significantly increased and remained elevated through 7 weeks [37]. However, consumption of a 45% HFD for 12 weeks led to a similar phenotype [38] (Table 2). 

*Rats*. Diets ranging from 45% to 62% fat caused adipocyte dysfunction and a T2D-like phenotype [41,43,44] (Table 2). Thirteen weeks of a 45% lard-based diet increased fat pad mass, adipocyte size and inflammation, but markers of T2D were not reported [41]. Diets of ~60% fat induced similar characteristics of adipose dysfunction with insulin and glucose intolerance, despite different fat sources [43,44]. Specifically, 62% mixed unsaturated fat caused significant adipose inflammation, accompanied by increased fasting glucose and homeostatic model assessment of insulin resistance (HOMA-IR) [44]. Diets composed of lower [41] and similar [43] percentages of fat from lard, reported comparable cellular and phenotypic changes in adipose. However, the duration of HFD consumption varied greatly in such studies, making it difficult to distinguish the importance of the fat concentration or source, from the duration of consumption (Table 2).

Changes in adipose integrity are influenced by sex, supporting the necessity for inclusion of female subjects. Following 11 weeks of a 57% HFD, only male rats developed oxidative stress and inflammation in subcutaneous adipose [42]. In female rats, increased subcutaneous and retroperitoneal fat mass was not associated with increased inflammatory cytokines or evidence of immune cell accumulation [42]. Notably, the source of fat and markers of T2D were not reported. However, in female mice, a similar diet increased adiposity without a change in glucose tolerance [40].

### 2.3. Pancreas 

*Mice*. The pancreas serves as a critical regulator of insulin and glucose homeostasis. Under low energy availability, glucagon could stimulate hepatic glucose production, while under energy surplus, insulin could facilitate glucose uptake. A better understanding of how different dietary models affect the pancreas will facilitate the development of a model that aligns with alterations in pancreatic function in the progression of human T2D. 

Six weeks of a 45% lard-based diet increased β-cell proliferation and mass, in the splenic region, contributing to the onset of insulin resistance in C57BL/6 male mice [45]. Following 12 weeks of the same diet, β-cell expression of adipose differentiation-related protein (ADFP) was increased, and pancreatic lipid accumulation was evident [46]. As a result, mice were hyperglycemic and hyperinsulinic. Human pancreatic β-cells treated with fatty acids showed a similar trend in ADFP expression [46]. 

In response to a 60% lard-based diet, reduced glucose tolerance and hyperinsulinemia were evident within 1 week, but insulin resistance did not manifest until 11 weeks [47]. At the onset, pancreatic cells compensated for increased glucose levels by doubling insulin secretion (detectable within 2 weeks), but insulin secretion became significantly impaired as insulin resistance developed and β-cell mass continued to increase [47]. Genes associated with hyperinsulinemia and β-cell proliferation were significantly elevated by week 8, which was 3 weeks prior to measurable insulin resistance [47]. More recently, a 60% HFD for 8 weeks, caused obesity, hyperglycemia, hyperlipidemia, and pancreatic β-cell hypertrophy in C57BL/6 mice [48] (Table 3). Metabolic profiling revealed that global metabolite changes of bioactive lipids associated with β-cell expansion and β-cell proliferation increased 1.75-fold [49]. 

*Rats*. Pancreatic inflammation in 60% HFD fed male Sprague–Dawley rats was associated with pancreatic atrophy, hyperinsulinemia, hyperglycemia, increased HOMA-IR, and pancreatic triglyceride (TG) accumulation following 24 weeks [50]. A similar dietary composition (66% fat), increased β-cell autophagy and glucagon production after 16 weeks [51] (Table 3). Elevated glucagon production is likely the cause for hyperglycemia reported at 16 weeks and at later time points in other studies [51,52]. However, 46% HFD for 12 weeks reduced glucose tolerance, indicating progression of the diabetic phenotype with a lower fat load [53]. Taken together, 46% and 66% HFD elicit β and α cell expansion, hyperinsulinemia, and reduced glucose tolerance [51,53]. Importantly, the source of fat in the aforementioned studies was not reported. Nonetheless, these similarities suggest that a fat content closer to a Western-based diet provides a sufficient stimulus to study HFD-induced pancreatic dysfunction.

Identifying the concentration and composition that best relates to pancreatic alterations in the human T2D population should be the priority, rather than adjusting the percentage of fat for the greatest tissue insult. Indeed, species-specific differences exist, such as primarily β-cell proliferation in rodents and β-cell apoptosis in humans prior to insulin resistance [49,54]. Therefore, if the pancreas is the tissue of interest, it may be beneficial to combine a moderate HFD model with low dose streptozotocin to reduce β-cell mass and allow for insulin levels to decline throughout T2D progression [55].

### 2.4. Brain

*Mice*. Chronic consumption of a HFD significantly impairs cognitive function by altering neuronal activity in the hippocampus of humans and rodents [57,58,59]. Altered hippocampal function is likely a result of chronic inflammation associated with obesity, with more severe outcomes from saturated fatty acids [59,60,61]. Furthermore, peripheral insulin and glucose homeostasis is largely regulated by hypothalamic insulin responsiveness [62]. Therefore, HFD-induced neuroinflammation, mitochondrial dysfunction, cognitive impairment, and whole-body glucose and insulin resistance highlight the brain as a critical organ in the pathogenesis of the T2D phenotype.

Lard-based diets of 45% and 60% increased the microglial and astrocyte activity in the hypothalamus, the primary regulatory region of energy balance [63,64]. Furthermore, both fat concentrations caused hyperinsulinemia, hyperglycemia, and reduced glucose tolerance, though this was more severe in response to 60% fat [63] (Table 4). Only the 60% HFD altered the hypothalamic, hippocampal, and cortex metabolite profile, showing a shift in cellular energy and metabolism. Similar metabolic dysfunction was reported following 4 weeks of HFD, accompanied by reduced insulin signaling in hypothalamic and hippocampal neurons [65] (Table 4). HFD also induces changes in the neuropeptide milieu to account for high nutrient density. In response to 60% HFD, the population of anorexigenic proopiomelanocortin (POMC) increased [64], and the expression of orexigenic neuropeptide-Y (NPY) decreased [66] (Table 4).

*Rats*. Impaired hypothalamic insulin signaling occurred following just three days of a low saturated fat diet (10% lard) in male Sprague–Dawley rats [67] (Table 4). The ability for hypothalamic insulin signaling to suppress white adipose lipolysis and hepatic glucose production was significantly impaired. Such hypothalamic insulin resistance was likely the cause of acute increases in plasma free fatty acids [67]. Diets slightly higher in saturated fats (20%) impaired learning and memory function when compared to standard diet (~4.5% fat) and diets higher in polyunsaturated fat (soybean oil) [70]. Following 18 weeks of a 39% HFD, Wistar rats developed glucose intolerance and increased body weight without hyperglycemia, hyperinsulinemia, or insulin resistance [71]. These rats displayed distinct neurometabolic alterations in the hippocampus and caudate-putamen, including glutamine and N-acetlylaspartate (NAA), two prominent amino acids in the brain that play a role in energy metabolism via lipid turnover and the citric acid cycle [52,71,72]. These data do contradict previous studies that showed no alterations in hippocampus metabolic profiles, particularly in genetically modified obese rats [73]. Male Wistar–Han rats fed a 40% HFD for 20 weeks demonstrated reductions in glucose tolerance in both plasma and cerebrospinal fluid following glucose challenge, suggesting that HFD induced changes in both central and peripheral glucose tolerance [68] (Table 4).

Six months of a 60% lard and soybean oil-based HFD impaired insulin-mediated microvascular perfusion and hippocampal cognitive function in Sprague–Dawley rats [74]. A similar response was reported in C57BL/6J mice [63,66,75]. 

Collectively, the literature supports moderate (45%) and high (60%) saturated fat concentrations as perturbing central and peripheral glucose and insulin sensitivity. While a 60% diet produces a more severe phenotype [63], we must consider whether the increased severity in a rodent model recapitulates the pathogenesis of T2D in humans. A 45% HFD, more in line with a Western-based diet, produces similar central and peripheral metabolic dysfunction, and may align more with the progressive development of human T2D.

### 2.5. Skeletal Muscle 

*Mice*. Chronic HFD consumption results in intramyocellular lipid storage in skeletal muscle, one of the largest contributors to glucose disposal in the body [24,74,76]. Excess intramyocellular lipid storage, particularly in sedentary populations promotes DAG accretion, protein kinase C activity, AMP-activated protein kinase (AMPK) inhibition, and attenuated glucose uptake [77,78]. The amount of saturated fatty acid in HFD differentially effects lipid deposition within skeletal muscle, potentially promoting differences in metabolism and oxidative capacity.

When consumed for 8 weeks, a 45% cocoa butter (saturated fat), olive oil (monounsaturated fat), or palm oil (a combination of fats), but not safflower oil (polyunsaturated fat)-based HFD, increased TAG and DAG content in skeletal muscle of C57BL/6J mice [19] (Table 5). All cohorts, except the 45% olive oil, demonstrated similar reductions in systemic glucose clearance, despite no changes in insulin-glucose transporter type 4 (GLUT4) expression [19]. β-oxidative remained unchanged, suggesting lipid accumulation occurred without a compensatory increase in oxidation [19]. 

One week of a 60% lard-based HFD reduced carbohydrate metabolism within skeletal muscle without insulin resistance [49]. When consumed for 15 to 20 weeks, a 60% lard-based diet causes significant dysfunction within skeletal muscle [6,14,30]. Such a diet caused skeletal muscle lipid accumulation, increased fasting glucose levels, and increased peroxisome proliferator-activated receptor gramma coactivator 1alpha (PGC-1α) protein expression [24]. Elevated PGC-1α expression is a characteristic of lipid accumulation in human muscle cells [79]. Furthermore, 191 skeletal muscle genes were altered following 20 weeks of the 60% lard-based HFD, favoring adipogenesis [6]. In agreement, 8 weeks of a similar diet reduced fatty acid oxidation and mitochondrial function [80] (Table 5). In human skeletal muscle, ectopic lipid accumulation, led to insulin resistance [81,82], similarly to that observed in rodent studies [19,24,83]. Subcellular localization of DAG in human skeletal muscle suggests that mitochondrial DAG is elevated in individuals with low insulin sensitivity and may be contributing to alterations in mitochondrial function [84]. 

Female mice are seldom studied in metabolic research; however, it is naive to assume that male and female rodents respond similarly to HFD models. A 58% coconut oil-based diet caused obesity only in male mice [85]. While muscle insulin sensitivity was reduced in both sexes, only males showed reduced whole-body glucose and insulin tolerance [85] (Table 5). 

*Rats*. Within a short exposure to 60% HFD, skeletal muscle metabolism and function are altered. These effects are fiber-type specific with increased lipid accumulation in type I and II intermediate fibers and reduced insulin-stimulated glucose uptake in a spectrum of type II fibers [88,89,90,91] (Table 5). Increased lipid accumulation in the soleus is associated with increase inflammatory signaling, evidenced by changes in IL-6 mRNA, and decreased control of oxidative stress [88].

A moderate 45% HFD also increased soleus lipid accumulation, as determined after 15 weeks [87]. Lipid accumulation was supported by increases in plasma membrane fatty acid binding protein (FABPpm), fatty acid transporter protein 1 (FATP1), fatty acid transporter protein 4 (FATP4), and CD36 [87] (Table 5). This diet was also associated with significant changes in whole-body insulin and glucose homeostasis. Hyperglycemia, hyperinsulinemia, and increased HOMA-IR were evident by 8 weeks of dietary consumption and persisted for 15 weeks [87]. Therefore, these data support the suitability of a macronutrient composition more closely aligned to a Western-based diet for studying the effects of HFD on skeletal muscle metabolism.

## 3. Choosing the Most Appropriate Model

As the percentage of fat in a diet increases, the metabolic changes become more significant, demonstrated by a 60% HFD animal model decreasing glucose sensitivity more rapidly (within one week) than a 45% HFD [92]. Furthermore, using a fat percentage that is 1.7-fold greater than a typical Western diet (~35% HFD) may induce pathology that is not characteristic of the T2D-like phenotype found in humans. Challenges with data interpretation and translatability to human studies include animal strain-specific responses, dietary variation, and the metabolic differences between humans and murine models [93]. A rodent’s heart rate ranges from 350–550 beats·min^−1^, but a human’s resting heart is approximately 70 beats·min^−1^ [93]. A mouse’s basal metabolic rate is ~7.5-fold greater than a human’s, suggesting the metabolic demand on a mouse is significantly greater [94]. Hepatic glycogen storage in a mouse is depleted every 16–24 h, and gluconeogenesis provides the majority of endogenous glucose, demonstrated by over 80% of glucose supplied via gluconeogenesis following a 4-h fasting period [94,95]. In contrast, humans utilize hepatic glycogen at approximately half that rate, even after an overnight (~12 h) fasting period [94]. Due to the faster depletion of glycogen stores and increased metabolic rate, mice consumed food and water more frequently throughout a 24-h period than humans. On average, mice consumed ~4.5 g food across 36 feedings and ~5.8 mL of water across 32 drinking times per day [96]. Feeding habit variability between rodents and humans may also contribute to dissimilarities in metabolism. As nocturnal animals, rodents consume the majority of their food in the evenings, but humans primarily eat during the day [97]. Further complicating this comparison is the treatment of rodents as diurnal rather than using a reverse light cycle to create a more natural rodent living environment. Circadian clocks are involved in many biological processes, including glucose metabolism and insulin secretion, particularly within pancreatic β-cells [98,99,100]. Disruptions in circadian rhythms could alter disease pathology or data interpretation, making translatability further challenging. Obesity and insulin resistance induced by a 60% high-fat diet caused alterations in circadian rhythm patterns in the hypothalamus [101]. Exogenous dopamine administration during appropriate peaks of circadian rhythm stimulated a physiological response mirroring a non-obese and an insulin sensitive rodent [101].

**Figure 1:**
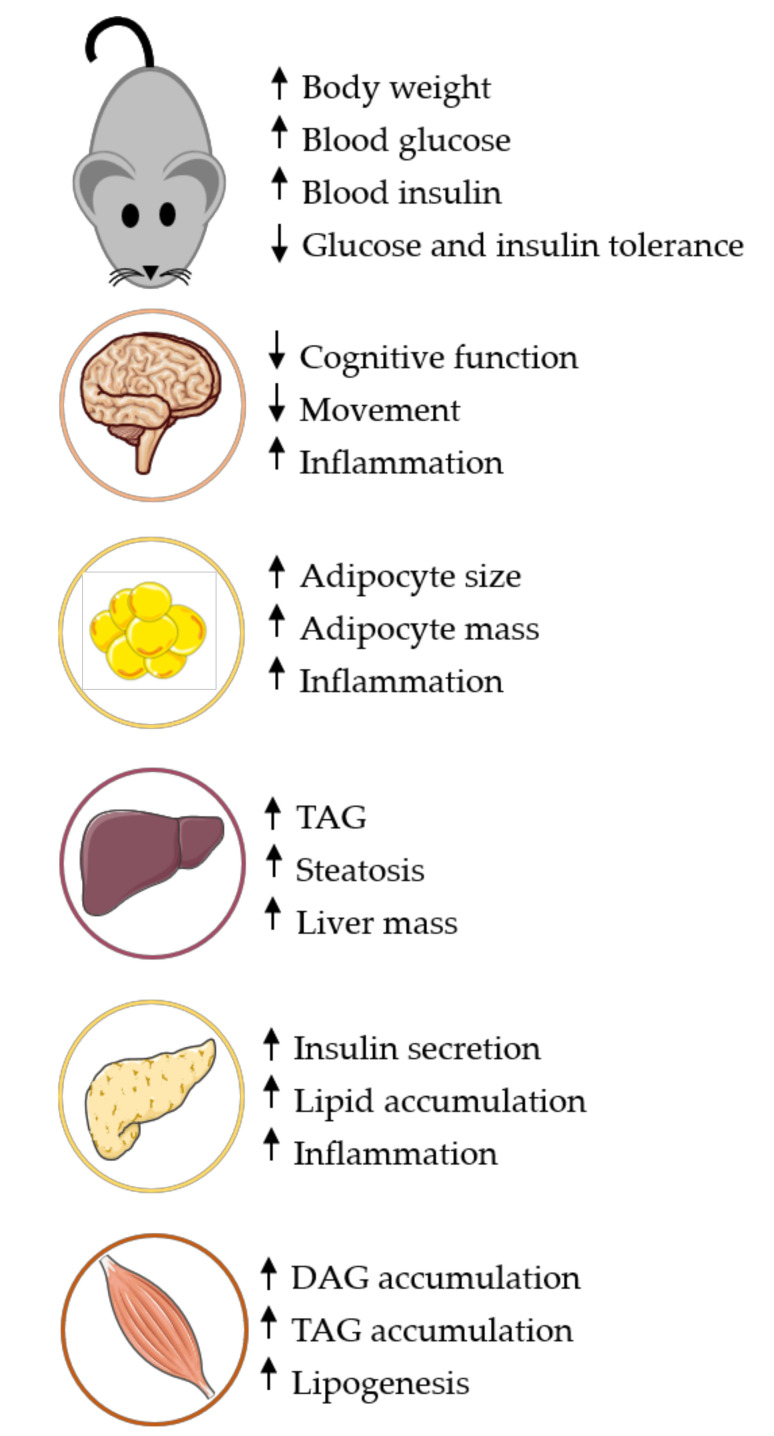
Rodent tissue responses to Western-like diet. A 45% HFD provides a progressive development of tissue and whole-body changes consistent with the T2D-like phenotype. Using higher fat percentages may create a greater magnitude of tissue dysfunction or accelerate the process, resulting in missed opportunity to study key aspects of disease progression. Detailed tissue responses could be found in Table 1 through Table 5. High-fat diet (HFD), Type 2 Diabetes (T2D), Triacylglycerol (TAG) and Diacylglycerol (DAG).

The full etiology of T2D cannot be accurately assessed and entirely translated to clinical practice because rodents experience β-cell proliferation or increased β-cell mass, rather than the loss of β-cell mass or β-cell failure [54]. To most effectively translate findings to the human population, an optimal animal model or a combination of a few select models may be most appropriate. Although the Western diet is known for excess fats, carbohydrates, and sugars, a longitudinal study over 17 years concluded that less than 35% of the average adult’s diet actually comes from fats [102]. According to the National Health and Nutrition Examination Survey, the mean dietary intake for adults (≥20 years) is composed of ~16% protein, ~47% carbohydrates, and ~35% fat. Nearly one-third of the total fat intake is saturated fat, demonstrating that there is a combination of fat sources in the typical Western diet. This data suggests that including a variety of fat sources (saturated, monounsaturated and polyunsaturated fats) would more accurately mimic the diet macronutrient composition seen in the average population. Based on this literature review, a 45% HFD with a combination of fat sources (monounsaturated, polyunsaturated, and saturated fats) induces pathological changes in rodents that closely resembles the T2D-like phenotype etiology in human populations. For example, adipose tissue becomes inflamed with a 45% high-fat model, but adipose tissue undergoes severe inflammation, remodulation, and adipocytosis following a 60% high-fat diet [35,103]. Commonly used 60% HFDs also contain more than nine times the amount of saturated fat (lard) when compared to unsaturated fat (soybean oil), suggesting that there are fat composition variables that need continued refinement to improve mirroring the human diet composition and translatability to target populations. Based on the literature reviewed here, we propose a 45% HFD composed of one-third saturated fats and two-thirds unsaturated fat closely aligns with the typical human Western diet and disease progression (Figure 1). Utilizing such a composition may provide better alignment between rodent and human T2D phenotypes and more inter-study agreement. 

It is important to acknowledge limitations to this review. While we attempted to use an un-biased review of the literature that used non-genetically altered rodents, we were not able to include all relevant publications. Those chosen provided sufficient methodological detail regarding dietary composition, intervention duration, and comprehensive sets of data. Moreover, only studies that compared HFD to a standard diet groups were included. Those in which the HFD served as the control against a pharmacological treatment were excluded. Mouse studies were focused on the C57BL/6 background because it is the most common. However, because the inclusion for female mice was rare, we included studies that investigated sexual dimorphism using other strains. Sprague–Dawley and Wister strains were targeted for rat studies because of their predominance in the literature. We attempted to narrow our literature to the last 5 years, particularly for studies included in the tables; however, in some cases we had to expand our search to accommodate for our inclusion criteria. Furthermore, this review focuses exclusively on HFD composition and concentration. Other dietary concerns, such as sugar, cholesterol, fiber, vitamin, and mineral composition and their combination with pharmacological strategies should be considered but are beyond the scope of this review. Additionally, numerous transgenic models exist, such as Ob/Ob, db/db, and KKAy, which cause metabolic dysfunction. Likewise, genetic models alone and in combination with dietary and pharmacological intervention are commonly used, particularly in the study of central insulin and glucose homeostasis. Lastly, animal models often include one gender, typically males, to avoid complications with the estrous cycle in female rodents, but diet-induced animal models can exhibit sexual dimorphisms [104]. Future studies should make every effort to include male and female rodents to comprehensively enhance our understanding of HFD models of T2D and associated phenotypes. 

## Figures and Tables

**Table 1:** Liver.

Source	Fat Source	Macronutrients (% kcal)	Duration	Strain; Sex	Tissue Findings	T2D Status
[19]	(a) Cocoa butter (b) Palm oil (c) Olive oil (d) Safflower oil	45% fat 20% protein 35% carbohydrate All diet formulations maintained same ratio	8 weeks	C57BL/6 mice; Male	↑ Liver TAG in palm and olive oil groups	↑ Body weight in palm oil compared to cocoa butter. ↓ Glucose tolerance: cocoa, palm and safflower oils
[6]	Hydrogenated coconut oil	59% fat 15% protein 26% carbohydrate	20 weeks	C57BL/6 mice; Male	↑ FABP mRNA ↑ Inflammation ↑ Increased BTNL2	↑ Fat mass ↓ Insulin sensitivity ↑ Plasma insulin
[22]	Lard and Soybean oil	60% fat 20% protein 20% carbohydrate	20 weeks	C57BL/6 mice; Female	↑ Lipid accumulation ↑ Hepatic steatosis	↑ Body weight ↓ Glucose tolerance ↑Serum insulin ↑ Serum glucose
[20,21,23,24]	Lard and Soybean oil	60% fat ~20% protein ~20% carbohydrate	8 weeks [23] 15 weeks [24] 18 weeks [20] 20 weeks [21]	C57BL/6 mice; Male	↑ Inflammation ↑ Lipid accumulation ↑ Fibrosis	↑ Body weight ↑ Serum glucose ↑ Serum insulin ↑ HOMA-IR
[25]	Lard	45% fat 30% protein 25% carbohydrate	4 weeks	Wister rats; Male	↑ Liver mass ↑ Liver TAG ↑ Hepatic steatosis	↑ Body weight ↓Insulin sensitivity ←→ Plasma glucose
[26]	Lard and Soybean oil	45% fat 20% protein 35% carbohydrate	12 weeks	Sprague-Dawley rats; Male	↑ Lipid accumulation ↑ Lipogenic gene and protein expression	↑ Body weight
[27]	Butter	58% fat 25% protein 17% carbohydrate	18 weeks	Sprague-Dawley rats; Male	↑ Inflammation ↑ Lipid accumulation ↓ Insulin signaling ↑ Hepatic necrosis ↑ Oxidative stress	↑ Body weight ↑ Serum glucose ↑ Serum insulin ↑ HOMA-IR

Studies cited in this table were limited to those that included macronutrient composition. The direction of arrows indicate change when compared to a standard diet control group within that study. Triacylglycerol (TAG), butyrophilin-like Protein 2 (BTNL2), fatty acid binding protein (FABP), major histocompatibility complex I and II (MHC I and II), and homeostatic model assessment of insulin resistance (HOMA-IR).

**Table 2:** Adipose.

Source	Fat Source	Macronutrients (% kcal)	Duration	Strain; Sex	Findings	T2D Status
[38]	Lard and Soybean oil	45% fat 20% protein 35% carbohydrate	12 weeks	C57BL/6; Male	↑ Body weight ↑ Epididymal fat mass and adipocyte size ↑ Inflammation	↑ Body weight ↑ Blood glucose
[33]	(1) Palm oil (2) Sunflower oil	(1) 45% fat 20% protein 35% carbohydrate (2) 45% fat 20% protein35% carbohydrate	24 weeks	C57BL/6; Male	↓ SFA: Adipose insulin signaling ↑ SFA: Inflammation ↑ SFA: Adipocyte size ↑ SFA and MUFA: Epididymal, visceral, subcutaneous, and perirenal fat pad mass	↓ Palm and sunflower oils: Glucose tolerance ↑ Sunflower oil: Hyperinsulinemia ↑ Palm oil: Hyperinsulinemia above sunflower oil
[37,39]	Lard and Soybean oil	60% fat 20% protein 20% carbohydrate	7 to 8 weeks	C57BL/6; Male	↑ Subcutaneous fat ↑ Visceral fat ↑ Adipocyte size ↑ Lipogenic gene expression	↑ Body weight ↑ Serum glucose
[40]	Lard and Soybean oil	60% fat 20% protein 20% carbohydrate	12 weeks	C57BL/6; female	↑ Perirenal fat ↑ Gonadal fat ↑ Mesenteric fat	↑ Body weight ←→ Glucose tolerance
[35]	Lard and Soybean oil	60% fat 20% protein20% carbohydrate	20 weeks	C57BL/6; Male	↑ Adipose weight ↑ Adipocyte size peaks at 12 weeks ↑ Adipocyte death peaks at 16 weeks ↑ Inflammation	↑ Body weight ↑ Serum insulin by 8 weeks ↑ Insulin resistance by 8 weeks ↑ HOMA-IR by 8 weeks
[41]	Lard and Soybean oil	45% fat 20% protein 35% carbohydrate	13 weeks	Sprague-Dawley rats; Male	↑ Retroperitoneal and epididymal fat mass ↑ Adipocyte size ↑ Adipogenic gene expression ↑ Macrophage accumulation ↑ Inflammation	↑ Body weight
[42]	Unknown	57% fat 10% protein 31% carbohydrate	11 weeks	Wister rats; Male and Female	↑ Subcutaneous and retroperitoneal fat mass and adipocyte diameter in males and females ↑ Oxidative stress in males only ↑ Subcutaneous fat inflammation in males only	↑ Body weight males only
[43]	Lard and Soybean oil	60% fat 20% protein 20% carbohydrate	8 weeks	Wister rats; Male	↑ Ceramide content ↑ DAG content ↑ Plasma free fatty acids	←→ Body weight ↑ Plasma glucose ↑ Plasma insulin ↑ HOMA-IR
[44]	UFA Mix: Sheep rump fat	62.1% fat 16% protein 28.2% fat	20 weeks	Wister rats; Males	↑ Inguinal, mesenteric, epididymal, retroperitoneal, and perirenal fat mass ↓ Capillary density ↑ Increased macrophage crown-like structures	↑ Body weight ↑ Blood glucose ↑ HOMA-IR ←→ Insulin

Studies cited in this table were limited to those that included macronutrient composition. The direction of arrows indicate change when compared to a standard diet control group within that study. Homeostatic model assessment of insulin resistance (HOMA-IR), saturated fatty acid (SFA), monounsaturated fatty acid (MUFA, and diacylglycerol (DAG).

**Table 3:** Pancreas.

Source	Fat Source	Macronutrients (% kcal)	Duration	Strain; Sex	Findings	T2D Status
[45]	Lard and Soybean oil	45% fat 20% protein 35% carbohydrate	6 weeks	C57BL/6; Male	↑ Beta cell proliferation in splenic region ↑ Increased insulin secretion from isolated islets	↑ Body weight ↑ Plasma insulin ↓ Glucose tolerance ↓ Insulin tolerance
[46]	Lard and Soybean oil	45% fat 20% protein 35% carbohydrate	12 weeks	C57BL/6; Male	↑ Lipid accumulation in acinar cells ↑ Adipose differentiation-related protein (ADFP)	↑ Body weight ↑ Serum insulin ↑ Blood glucose
[48]	Lard and Soybean oil	60% fat 20% protein 20% carbohydrate	8 weeks	C57BL/6; Male	↑ Islet size ↑ Islet Insulin	↑ Body weight ↑ Blood glucose ↑ HbA1c ↑ Serum Insulin ↑ HOMA-IR
[53]	Not reported	46% fat 20.3% protein 24% carbohydrate	12 weeks	Sprague-Dawley rats; Male	↑ Mast cell accumulation ↑ Islet area and proliferation ↑ β and α cell area	↑ Plasma insulin ↓ Glucose tolerance
[56]	Not reported	60% fat 18% protein 22% carbohydrate	8 weeks	Sprague-Dawley rats; Male	Glucose-stimulated islet insulin secretion	Glucose tolerance
[51]	Not reported	66.43% fat 18.08% protein 15.48% carbohydrate	8 and 16 weeks	Sprague-Dawley rats; Male	↑ Islet cell insulin at 8 and 16 weeks ↑ Glucagon at 16 weeks ↑ β and α cell area at 16 weeks ↑ β cell autophagy at 16 weeks	↑ Body weight at 16 weeks ↑ Plasma glucose at 16 weeks ↑ Serum insulin at 8 and 16 weeks ↑ HOMA-IR at 8 and 16 weeks

Studies cited in the table were limited to those that included macronutrient composition. The direction of arrows indicate change when compared to a standard diet control group within that study. Homeostatic model assessment of insulin resistance (HOMA-IR) and hemoglobin A1c (HbA1c).

**Table 4:** Brain.

Source	Fat Source	Macronutrients (% kcal)	Duration	Strain; Sex	Findings	T2D Status
[63]	Lard and soybean oil	10, 45, or 60% fat 20% protein 70, 35, or 20 % carbohydrates	24 weeks	C57BL/6; Male	45% and 60% fat ↓ Spontaneous activity ↓ Locomotion ↑ Neuroinflammation 60% fatAltered metabolite profile	45% and 60% fat ↑ Body weight: 60% > 45% ↑ Plasma glucose: 60% > 45% ↑ Plasma insulin ↑ Plasma Leptin ↓ Glucose tolerance: 60% > 45% at 2 h
[65]	Lard and soybean oil	60% fat 20% protein 20% carbohydrates	4 weeks	C57BL/6; Male	↓ Insulin signaling in isolated hypothalamic and hippocampal neurons ↓ Mitochondrial function ↑ Oxidative stress	↑ Body weight ↑ Fat mass ↑ Plasma glucose ↑ Plasma insulin ↑ HOMA-IR
[66]	Palm oil	60% fat 16% protein 24% carbohydrates	8 weeks	C57BL/6; Male	↓ Socialization behavior ↑ Disruption of normal circadian feeding Hypothalamic NPY expression	↑ Body weight
[64]	Lard and soybean oil	60% fat 20% protein 20% carbohydrates	12 weeks	C57BL/6; Female	↑ Microglia in hypothalamic arcuate nucleus ↑ Trend in neurogenesis of POMC neurons	↑ Body weight ↑ Fat mass
[67]	Lard	10 % added to standard diet	3 days	Sprague-Dawley Rats; Male	↓ Hypothalamic insulin sensitivity ↓ Hypothalamic insulin-stimulated adipose lipolysis ↓ Hypothalamic insulin-stimulated hepatic glucose production	←→ Body weight ←→ Plasma glucose ←→ Plasma insulin
[68]	Not reported	45% fat 20% protein 35% carbohydrates	20 weeks	Wistar-Han Rats; Male	←→ CSF glucose ↓ CSF glucose tolerance	↑ Body weight Plasma glucose ↓ Glucose tolerance
[69]	Lard and soybean oil	60% fat 20% protein 20% carbohydrates	24 weeks	Sprague-Dawley Rats; Male	↓ Cognitive function ↓ Insulin-stimulated hippocampal perfusion ↓ Hippocampal insulin signaling ↓ Hippocampal eNOS	↓ Whole body glucose disposal ↑ Plasma insulin

Studies cited in the table were limited to those that included macronutrient composition. The direction of arrows indicate change when compared to a standard diet control group within that study. Cerebrospinal fluid (CSF), proopiomelanocortin (POMC), endothelial nitric oxide synthase (eNOS), and homeostatic model assessment of insulin resistance (HOMA-IR).

**Table 5:** Skeletal Muscle.

Source	Fat Source	Macronutrients (%kcal)	Duration	Strain; Sex	Findings	T2D Status
[19]	(a) Cocoa butter (b) Palm oil (c) Olive oil (d) Safflower oil	45% fat 20% protein 35% carbohydrateAll diet formulations maintained same ratio	8 weeks	C57BL/6; Male	↑ Gastrocnemius TAG and DAG: cocoa butter, palm oil, and olive oil	↑ Body weight in palm oil compared to cocoa butter. ↑ Glucose tolerance: cocoa, palm and safflower oils
[85]	Coconut oil and soybean oil	58% fat 17% protein 25% carbohydrate	16 weeks	FVB; B6;Male and Female	↓ Muscle insulin sensitivity: Males and Females	↑ Body weight: Males only ↑ Plasma glucose: Males only ↓ Glucose and insulin tolerance: Males only
[6,80]	Hydrogenated coconut oil	59% fat 15% protein 26% carbohydrate	8 weeks [83] 20 weeks [6]	C57BL/6; Male	↑ Fatty acid transport ↑ Lipogenesis ↑ Muscle Adipocyte differentiation ↓ Fatty acid oxidation ↓ Mitochondrial function	↑ Body weight ↑ Blood glucose ↑ Plasma insulin ↓ Insulin sensitivity
[86]	Lard and soybean oil	60% fat 20% protein 20% carbohydrate	15 weeks	C57BL/6; Male	↓ Insulin sensitivity ↑ Muscle weight ↑ Inflammatory mRNA profile	↑ Body weight ↑ Serum insulin ↕ Glucose tolerance
[87]	Lard	45% fat 20% protein 35% carbohydrate	15 weeks	Sprague-Dawley rats; Male	↑ Soleus lipid accumulation ↑ Protein and mRNA supporting fatty acid transport and lipogenesis	↑ Body weight ↑ Plasma glucose ↑ Serum insulin ↑ HOMA-IR ↓ Insulin sensitivity
[88,89]	Lard and soybean oil	60% fat 20% protein 20% carbohydrate	2 weeks	Wister rats; Male	←→ Soleus and extensor digitorum longus weight ←→ Soleus and extensor digitorum longus insulin signaling ↓ Soleus force production ↓ Soleus glutathione ↑ Soleus IL-6 mRNA ↑ Lipid droplet size in soleus ↑ Percentage of large lipid droplets in soleus ↑ Lipogenic mRNA in soleus	←→ Body weight
[90,91]	Lard and soybean oil	60% fat 20% protein 20% carbohydrate	2 weeks	Wister rats; Male	↓ Glucose uptake by isolated type IIAX, IIX, IIBX, and IIB fibers ↑ Lipid droplet density in type I, IIA, and IIAX fibers ↑ Lipid droplet size in type I and IIA fibers ↓ Insulin-stimulated glucose uptake in whole muscle ↓ Insulin-stimulated glucose uptake in isolated type IIA, IIAX, and IIB fibers	←→ Body weight

Studies cited in the table were limited to those that included macronutrient composition. The direction of arrows indicate change when compared to a standard diet control group within that study. Diacylglycerol (DAG), triacylglycerol (TAG), interleukin 6 (IL-6), and homeostatic model assessment of insulin resistance (HOMA-IR).
